# Dynamic In-Plane Compression and Fracture Growth in a Quasi-Isotropic Carbon-Fiber-Reinforced Polymer Composite

**DOI:** 10.3390/ma17246296

**Published:** 2024-12-23

**Authors:** Yogesh Kumar, Mohammad Rezasefat, Zahra Zaiemyekeh, Haoyang Li, Patricia Dolez, James Hogan

**Affiliations:** 1 Department of Mechanical Engineering, University of Alberta, Edmonton, AB T6G 2R3, Canada; yogesh3@ualberta.ca (Y.K.); rezasefa@ualberta.ca (M.R.); zaiemyek@ualberta.ca (Z.Z.); haoyang@ualberta.ca (H.L.); 2 Department of Human Ecology, University of Alberta, Edmonton, AB T6G 2R3, Canada; pdolez@ualberta.ca

**Keywords:** highstrain rate, carbon-fiber-reinforced polymer, digital image correlation, in-plane compression, crack speed, fracture plane

## Abstract

This study presents an experimental investigation of the quasi-static and dynamic behavior of a quasi-isotropic carbon-fiber-reinforced composite subjected to in-plane compressive loading. The experiments were performed at strain rates ranging from $4\times 10^{-5}$ to ∼1200 $s^{-1}$ to quantifythe strain-rate-dependent response, failure propagation, and damage morphology using advanced camera systems. Fiber bridging, kink band formation, dominance of interlaminar failure, and inter-fiber failure fracture planes are evidenced through post-mortem analysis. The evolution of the in-plane compressive strength, failure strength, and stiffness are quantified across the strain rates considered in this study. For an in-depth understanding of the failure propagation, crack speeds were determined in two subsets; (i) primary and secondary cracking, and (ii) the interfaces participating in the crack propagation. Lastly, a modified Zhu–Wang–Tang viscoelastic constitutive model was used to characterize the dynamic stress-strain and compressive behavior of the quasi-isotropic composite under in-plane compression.

## 1. Introduction

Carbon-fiber-reinforced polymer (CFRP) composites are known for their high weight-to-strength ratio, enhanced thermal and mechanical properties, corrosion resistance, and higher impact energy absorption capabilities compared to traditional metallic materials [1,2,3,4]. Over the past decades, these advanced materials have been widely adopted as components within structures in defense [5], automotive [6], aerospace [7], and civilian applications [8], as the manufacturing process allows for tailoring the finished material as per the requirements [9,10]. The applications to different domains exhibit different loading conditions including shock, crash, ballistics, and fatigue [11,12,13,14,15]. This variation in the time-dependent loading conditions necessitates the evaluation of the strain-rate-dependent material behavior. Sierakowski [16] comprehensively reviewed the various experimental techniques that can be utilized towards exploring the strain-rate-dependent behavior of the filamentary composite materials at different strain-rate levels ($10^{-8}–10^{6}$ $s^{-1}$). The size and geometry of the samples considered in such high strain rate experiments did not have any statistical effects on the measured properties of the material [17]. Ninan et al. [18] studied several factors (friction, pulse shapers, and loading faces) and provided protocols for effective and accurate conduction of the experiments. The overall resistance of the material to dynamic loading is dependent on the visco-plastic nature of the matrix, fiber orientation, and relative alignment with the loading direction, and whether it is subjected to in-plane, out-of-plane, or off-axis loading conditions [19,20,21,22]. Recently, many articles have characterized the dynamic response of fiber-reinforced composites aimed at studying the effect of fiber orientations [23], constituents of the materials [24], environmental conditions [25], and loading direction [26]. However, most of the studies focused on unidirectional stacking of plies or are limited to the qualitative examination of the failure features. Unlike unidirectional composites, which exhibit highly anisotropic responses, quasi-isotropic composites feature a symmetric ply arrangement ([0°, ±45°, 90°]s) designed to deliver more uniform mechanical properties across all in-plane directions. This layup improves the composite’s resistance to delamination and interlaminar shear while preserving substantial strength and stiffness. Nevertheless, the damage mechanisms and failure modes in quasi-isotropic laminates remain less explored, particularly under compressive loading. Xie et al. [26] studied the compressive behavior of a unidirectional carbon/epoxy laminate in all three principal directions (fiber, transverse, and thickness) for quasi-static and high strain rate loading. They showed that the damage kinetics and failure modes are strain-rate-dependent along with the compressive strength and the failure strain observed during the experiments [27]. Delamination and longitudinal cracking were observed to be the dominating failure modes in the case of loading along the fiber direction, whereas loading in other principal directions led to matrix shearing and debonding [28,29,30]. Failure initiation and propagation in the laminated composites are complex phenomena as the driving failure mode varies with the configuration of the material system and loading conditions [31,32]. Inter- and intra-laminar, and inter-fiber failure are leading failure phenomena in laminated composites. Rezasefat et al. [33,34] presented a fast approach towards the determination of the Puck’s 3D inter-fiber failure fracture angle in unidirectional composites and later exercised the proposed model for a low-velocity impact case study. Guo et al. [35] proposed a failure function by differentiating the coefficients for the two transverse tension and compression states of the composite to develop the off-axis strength and failure envelope, motivated by the parabolic nature of the Puck failure criteria [36]. Validation of the proposed failure model was performed by limited experiments [37,38] and the classical theories [36,39].

The physics behind the failure initiation and propagation of fiber-reinforced composites varies with the nature and direction of the applied load with respect to the architecture of the material system. Arbauni et al. [40] performed high strain-rate compression experiments on woven composites to understand the damage mechanisms under out-of-plane and in-plane loading directions. The stress induced in the material was more severe during the in-plane loading condition compared to the out-of-plane one. Under out-of-plane loading, the damage was generated from the plastic deformation as the layers were crushed due to the shearing and development of micro- and macro-cracks. Subsequently, the damage was more catastrophic under in-plane compression loading as the damage propagated through the delamination of the layers and fiber buckling, as also concluded by similar studies [28,41,42,43]. Koerber and Camanho [41,44,45] explored the in-plane dynamic compressive behavior of an unidirectional IM7/8552 composite through experimental and analytical studies. However, the different results produced by the research team were only limited to the strain rate of 350 $s^{-1}$. It is worth noting that authors in [45] have also determined the fracture plane angle under transverse and various off-axis compression tests for the development of the quasi-static and dynamic yield stress, and failure envelope. Li et al. [46] experimentally investigated the different stacking sequences of a CFRP composite at quasi-static and high strain rate in tensile loading. The quasi-isotropic, cross-ply, and angle-ply samples exhibited very different responses under the same strain-rate loading. The stiffness was reported to be highly sensitive to the composite layup, as for the cross-ply and quasi-isotropic the magnitude decreased with increased strain rate, but the opposite trend was observed for the angle-ply samples. To sum up, there are very few studies that focus on the characterization and quantification of the failure propagation, especially under in-plane compression loading. The existing studies are also either limited to simple stacking sequences (unidirectional) or examined within the lower range of high strain rates.

Building on results reported in the literature, this study is focused on experimentally investigating the strain-rate-dependent behavior of a quasi-isotropic ${\left[0^{\circ }/\pm 45^{\circ }/90^{\circ }\right]}_{s}$ carbon-fiber-reinforced polymer composite laminate under in-plane compressive loading. The experiments were conducted at two quasi-static strain rates ($10^{-5}$ and $10^{-3}$ $s^{-1}$) using a servo-hydraulic 810 MTS machine and three high strain rates (∼300, ∼600, and ∼1200 $s^{-1}$) using a split-Hopkinson Pressure Bar coupled with a digital image correlation (DIC) system, as described in Section 2. The mechanical response, deformation mechanisms, and damage morphology during the experiments are presented and discussed in Section 3. Post-mortem analysis includes scanning electron microscope (SEM) imaging, and macro-scale-damaged samples and fragments from the experiments are also presented to aid in a better understanding of the failure modes and damage mechanisms. Due to the in-plane loading applied on the sample, the crack propagation between the different fiber orientations can be differentiated and measured. Crack speed measurements during the inter- and intra-laminar failure experienced by the sample are reported based on the primary and secondary cracking, and at different interfaces. Additionally, a non-linear visco-elastic Zhu–Wang–Tang (ZWT) analytical model is used to parameterize the constitutive behavior for the dynamic compression load case for the performed experiments.

## 2. Materials and Methods

In this section, a brief description of the material, sample preparation methodology, and experimental setup have been provided. Additionally, a description of the digital image correlation technique and validity of the dynamic experiments have been provided using the concept of stress-equilibrium factor [47].

### 2.1. Material Description and Sample Preparation

For this study, a quasi-isotropic unidirectional carbon-fiber-reinforced polymer $[0^{\circ }$/$\pm 45^{\circ }$/$90^{\circ }{]}_{s}$ composite cube samples have been used manufactured from flat panels. The constituents of the materials contain T700SC carbon fibers and toughened thermoset epoxy resin MTC510. The prepregs were cured using an autoclave process at 110 °C for 2 h with a constant pressure of 6 bar. The curing temperature was increased at the rate of 2 °C/min. The fiber volume fraction of the cured laminate was 60% with the measured cured ply thickness of 0.28 mm. The cubical shape of the samples aids in having a uniform flat speckle pattern, proven to be best for digital image correlation analysis, and for observing the fracture patterns and progression of failure modes within the different interfaces [48,49]. The composite panels of thickness 4±0.05 mm were further machined using a low-speed diamond saw with coolant to obtain the small cubical samples compatible with the split-Hopkinson Pressure Bar experimental setup. The manufacturing of these small samples was performed by Karma Machining & Manufacturing Ltd., Edmonton, AB, Canada. The samples were machined in two stages: firstly, the samples were clamped using two custom-built jigs to ensure accuracy, and achieve desired parallelism on the sample’s sides. Further, the samples were machined using a knee-style vertical milling machine, and an HSS slitting wheel with a cutting speed of 100 SFM (strokes-per-minute) and feedrate of $3\times 10^{-4}$ IPT (inches-per-tooth) and a constant supply of coolant (Fuchs EcoCool 411) to avoid the degrading of the matrix due to frictional heat. Later on, the faces of the samples were examined to ensure they were parallel and perpendicular to each other and if required, fine polishing was also performed with 320 grit sandpaper.

### 2.2. Mechanical Testing Setup

**Figure 1:**
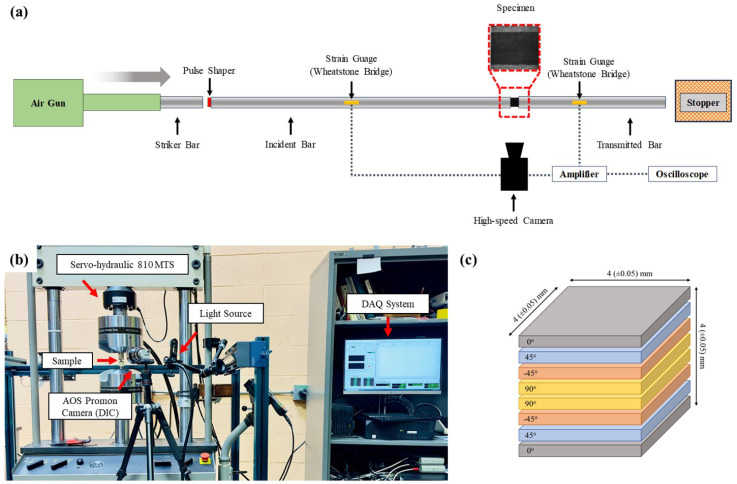
Schematic of (**a**) split-Hopkinson Pressure Bar (SHPB) utilized for high strain rate testing, (**b**) MTS machine for quasi-static compression testing, and (**c**) schematic of the carbon-fiber-reinforced polymer depicting the stacking sequence and dimension of the sample.

The quasi-static and dynamic compression testing of the quasi-isotropic composite samples were performed using a standard servo-hydraulic 810 MTS machine and split-Hopkinson Pressure Bar with different pulse shapers, as listed in Table 1. In both of the testing environments, a high-pressure grease was applied between the contact surface of the sample and the respective apparatus to eliminate the frictional effect and allow for free lateral motion [48]. Figure 1 shows the schematic of the experimental setup with details on the stacking sequence and dimensions of the sample studied in this work.

For quasi-static testing, a high-speed camera AOS PROMOS U750 with high-resolution images of 1280×1024 pixels at 5–100 frames per second (adjusted to the loading rate applied by the 810 MTS machine) was utilized to capture the full strain field with the DIC technique. The speckle pattern desired to obtain good data analysis for DIC was obtained using a fine-tipped airbrush with 0.15 mm nozzle diameter which produced a random speckle pattern with spot size in the range of 20–30 μm. The quasi-static uniaxial compression was applied using a displacement control setting to attain the strain rate in the order of $10^{-5}$ $s^{-1}$ and $10^{-3}$ $s^{-1}$. The force history was recorded through the 100 kN load cell on the MTS machine and the strain field was measured through 2D-DIC analysis, and was post-processed to obtain the strain-strain curve of the material. The experiments for each configuration of the strain rate were performed at least three times to show repeatability.

For the dynamic characterization of the material under uniaxial compression, the experiments were conducted on the SHPB apparatus with pulse shapers. Because of the dispersion effects, pulse shapers reduce the stress wave oscillation and help to achieve the desired stress equilibrium during the experiments [49]. Paper as a pulse shaper reduces the noise in the signals without degrading the amplitude of the wave, hence providing the highest strain rate induced in the sample [50]. High-Density Polyethylene (HDPE) is considered to be a reasonably good pulse shaper as it reduces the amplitude of the stress wave without affecting the shape [49,51]. In this study, three different configurations are used to achieve the strain rate range from ∼300–1200 $s^{-1}$, either by changing the pulse shaper and/or the applied pressure from the air gun. To replicate the shock loading, a striker bar (diameter: 12.7 mm and length: 304 mm) impacts the incident bar (diameter: 12.7 mm and length: 1016 mm) which transmits an elastic wave through the transmitted bar (diameter: 12.7 mm and length: 914 mm), resulting in a dynamic loading experienced by the sample. The bars are made of C-350 maraging steel with a yield strength of 2.36 GPa, an elastic modulus of 200 GPa, and a density of 8080 ${\mathrm{kg}/m}^{3}$. An HBM Gen3i high-speed recorder coupled with Bessel IIR pre-filter for filtering the low-frequency noise was employed for sampling the data at 100 MHz from the two strain gauges (Micro 184 Measurements CEA 13-250UN-350) attached to the incident and transmitted bars. For quasi-static compression testing, the 2D-DIC analysis requires an ultra-high-speed camera to trace the displacement of the fine-spotted speckle pattern for full-strain field maps. Shimadzu HPV-X2 ultra-high-speed camera with K2 DistaMax Infinity lens and LED ring light is equipped with the SHPB apparatus for capturing the whole event in 128 images, each with a resolution of 400×250 pixels recorded at 1 million frames per second. The experimental setup in this study was the same as in previous studies conducted by the author’s research group [48,49,50,51]. Further details on the theoretical background and functioning of the SHPB system have been provided in the literature [41,44,52,53,54].

### 2.3. Digital Image Correlation Analysis

In the present study, both experimental setups were equipped with two-dimensional DIC technology with the means of high-speed cameras and randomized fine speckle patterns on the samples. The cameras were able to capture high-resolution images during the deformation of the samples to post-process them in the VIC-2D V6 software from Correlated Solution Inc. (Irmo, SC, USA). The software performs pixel tracking on the fine spots over the samples to obtain relative displacement and further calculate the spatial and temporal histories of strain components [55]. The reference image for the analysis was considered from the first frame of the event. The surface of the sample was discretized into a subset of 23×23 pixels with a step size of 7 pixels during the analysis to ensure the eventual smoothness of the strain-time plots. A zero-normalized square sum of difference (ZNSSD) method with the optimized 8-tap interpolation scheme was utilized in the analysis. Before discussing the obtained results, it is important to establish the stress equilibrium condition achieved by the sample during the high strain rate experiments [25,56,57]. Ravichandran and Subhash [47] proposed a stress-equilibrium factor *R*(*t*) to judge the effectiveness of the high strain rate experiments by evaluating the stress states on the contact surface between the incident and transmitted bars, and the samples, given as

(1) $$R\left(t\right)=\frac{2|(\sigma_{1}\left(t\right)-\sigma_{2}\left(t\right)|}{|\sigma_{1}\left(t\right)+\sigma_{2}\left(t\right)|}$$

(2) $$\sigma_{1}=\frac{A_{b}E_{b}(\varepsilon_{I}\left(t\right)+\varepsilon_{R}\left(t\right)}{A_{s}}$$

(3) $$\sigma_{2}=\frac{A_{b}E_{b}\left(\varepsilon_{T}\left(t\right)\right)}{A_{s}}$$

where $A_{b}$ and $A_{s}$ are the cross-sectional areas of the bar and specimen, respectively. $E_{b}$ denotes the elastic modulus of the bar material. $\sigma_{1}$ and $\sigma_{2}$ are the stresses in the contact surfaces of the incident and transmitted bar calculated based on the incident pulse $\left(\varepsilon_{I}\right)$, transmission pulse $\left(\varepsilon_{T}\right)$, and reflected pulse $\left(\varepsilon_{R}\right)$ recorded by the mounted strain gauges.

**Figure 2:**
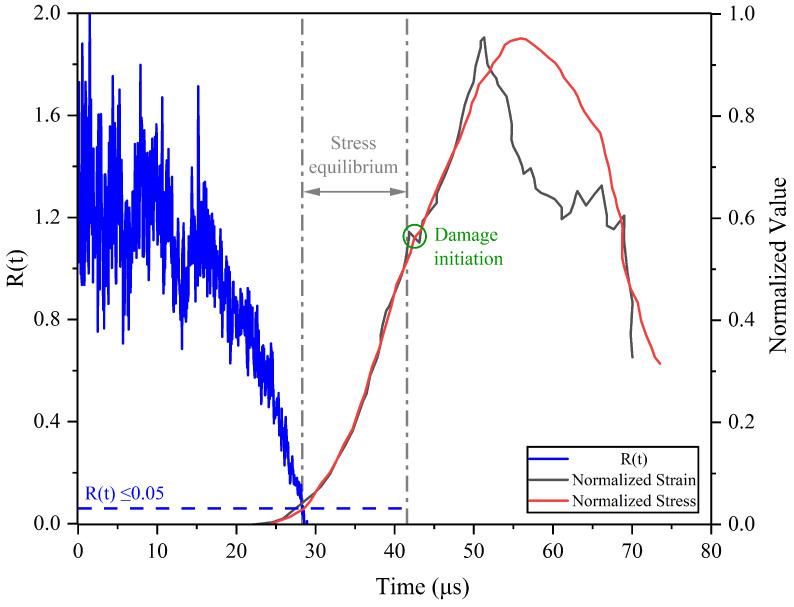
The strain- and stress-time plots along with the calculated stress-equilibrium factor for the strain rate 643.4 $s^{-1}$. The plot signifies the establishment of stress equilibrium as R(t) reaches less than 0.05 at 28 μs. The damage initiation at 42 μs indicates the loss of force equilibrium, as marked by the grey lines.

Figure 2 shows the stress and strain histories for the high-strain uniaxial compression of the quasi-isotropic composite sample with HDPE pulse shaper and applied pressure of 40 psi from the air gun to the striker. Initially, the stress wave was unable to propagate through the transmitted bar $\left(\sigma_{2}=0\right)$ resulting in R(t) = ∼2. However, after a few reflections of the stress wave through the sample, the stress equilibrium factor reaches R(t) = ≤0.05, symbolizing the establishment of the stress-equilibrium state at ∼28 μs [25,56]. The sample remains in the stress-equilibrium state until the damage initiation occurs at 42 μs. The overlapping of the strain- and stress-time plot between the beginning of stress equilibrium and initiation of damage in the sample also indicates the establishment of a good equilibrium, as shown in Figure 2. The slope is calculated at this aforementioned window on the strain-time plot to obtain the global strain rate experienced by the sample [48,50]. The slight shift between the peak failure strain and stress experienced by the sample is believed to be caused by evident fiber bridging within the different fiber orientations [58,59]. The reported stress-equilibrium check was performed on the experiment run of strain rate 643.4 $s^{-1}$.

## 3. Results

### 3.1. Mechanical Response and Strain-Rate-Dependent Behavior

**Figure 3:**
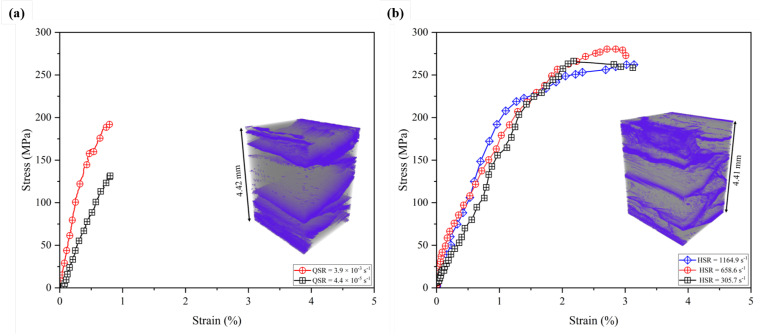
Stress-strain curve obtained from the in-plane compression testing under (**a**) quasi-static rate and (**b**) high strain rate testing. The shown representative CT-scans are from the strain rate $4.4\times 10^{-5}$ $s^{-1}$ for the quasi-static case, and 305.7 $s^{-1}$ for the higher strain rate case. The blue region shows the fracture network developed under the compression loading. The fracture propagated under quasi-static rate ($4.4\times 10^{-5}$ $s^{-1}$) tends to be more centralized at the interlaminar interfaces of the composite, due to the predominance of shear phenomenon from the relative orientation angle of the fibers. In the high strain rate case (305.7 $s^{-1}$), the fracture network is evident at both interlaminar and intralaminar interfaces.

In this section, the stress-strain plots obtained at quasi-static and dynamic strain rates of the quasi-isotropic composite under in-plane compression are presented, as shown in Figure 3. To ensure repeatability, each experiment case was performed three times, and the obtained results are quantified in Table 1.

The general trend of increasing compressive strength and failure strain with increased strain rate is observed, as also reported in the literature [19,42]. Under quasi-static loading, the average peak stress reaches 156.4 MPa, which is 48.6% lower than the average peak stress value reached during the dynamic experiments, and similarly, for the failure strain, there is a substantial difference of 136.5%. The failure strain for the quasi-static load cases is consistent with an average strain of 0.8%. The failure strain increases from 1.9% to 3.2% as the strain rate increases from 321.0 $s^{-1}$ to 1188.1 $s^{-1}$. This demonstrates the strain-rate-dependent behavior of the studied composite material [54,60]. From the high strain rate experiments, the stress-strain plots are presented in Figure 3b. The plot illustrates an initial linear elastic deformation led by a nonlinear behavior with increasing strain on the sample which exhibits sub-critical crack propagation. The compressive strength increases from 224.5 MPa to 289.2 MPa from 321.0 $s^{-1}$ to 658.6 $s^{-1}$. However, the strength is reduced to 259.2 MPa as the strain rate reaches 1188.1 $s^{-1}$. The instantaneous deformation in the material did not allow enough time for the dissipation of the kinetic energy in the matrix during the event [61,62]. This leads to a sudden increase in the adiabatic temperature within the samples and can affect the strength of the composite over 700–1000 $s^{-1}$ strain rate [26,63]. The stiffness for the quasi-static experiments increases with the increasing loading rate and this is consistent with the other literature [64,65]. The in-plane loading forces the separation of the different plies once they reach the failure strain, due to the involvement of the weak interfaces, as shown in the post-mortem CT-scan representative inset in Figure 3. It can be observed that high strain rate experiments induce significantly more damage compared to the quasi-static ones. Qualitatively, both CT-scans depict interlaminar and intralaminar fracture networks, along with inter-fiber failure at the 90° plies.

**Figure 4:**
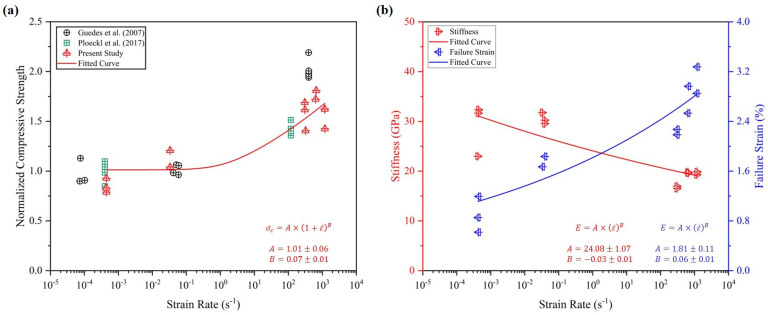
(**a**) The evolution of the compressive strength over the strain-rate spectrum, establishing the strain-rate-dependent nature of the composite, along with the data from the literature [44,66]. Similarly, (**b**) depicts failure strain and stiffness of the material versus strain rate. Curve fitting (equation inset in the figure) is carried out to better visualize the trends.

Figure 4 shows an evolution of the in-plane compressive strength, failure strain, and stiffness across the spectrum of strain rates. For the comprehensive representation of the strain-rate dependency of in-plane compressive strength and comparison with the existing literature [44,66], the values are normalized based on quasi-static strength. The data are fitted using a power law and the parameters are inset in Figure 4a. The failure strains and stiffness are plotted across the strain rates in Figure 4b. The failure strain increases with the increased strain rates, as also reported by [56,57]. However, the stiffness magnitude first increases within the quasi-static loading rates and then keeps degrading with the increased strain rates, where a similar trend for stiffness is reported by Gao et al. [64]. This is potentially caused by the viscoelastic nature and high sensitivity towards the strain rate of the employed thermoset matrix in the composite [67,68,69,70].

### 3.2. Damage Morphology and Failure Modes

The damage evolution process in the sample captured through advanced imaging systems allows the qualitative and quantitative understanding of dominating failure modes and interfacial physics in laminated composites. The micro defects (voids, inclusions, resin-rich zones, and weak interfaces) developed during the manufacturing process drive the damage evolution as the strain increases, and this results in a reduction in the stiffness of the material [26,71].

**Figure 5:**
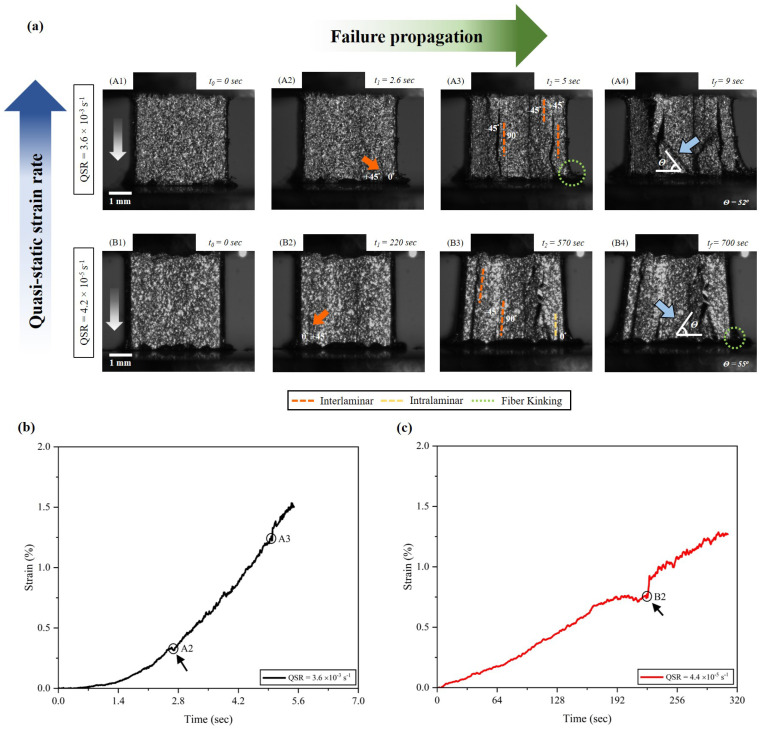
(**a**) Failure propagation in quasi-isotropic carbon-fiber-reinforced polymer composite under quasi-static strain rates (listed in the figure) loading. The white arrow in (**A1**) and (**B1**) indicates the loading direction for compression. (**A2**) and (**B2**) show the crack initiation in the sample at the interface of $0^{\circ }$/$45^{\circ }$ at corresponding times in the stress-strain plot in (**b**,**c**). In all the repetitions, we observed that the failure dominantly initiates at the $0^{\circ }$/$45^{\circ }$ interface, followed by the development of secondary cracks at the $-45^{\circ }$/$90^{\circ }$ interface, and ultimately leading to inter-fiber failure within the $90^{\circ }$ plies, as marked in (**A3**) and (**B3**), along with the $0^{\circ }$ plies experienced fiber kinking, as labeled in (**A3**) and (**B4**). The developed fracture planes are also highlighted in (**A4**) and (**B4**).

Figure 5 shows the failure propagation in quasi-isotropic carbon-fiber-reinforced composite under two quasi-static strain rates. The images from the high-speed camera are mapped out on the strain-time plot obtained from the DIC analysis. The failure dominantly initiates at the $0^{\circ }$/$+45^{\circ }$ interface for both quasi-static strain rates with the strain of 0.3% (marked as (A2) in Figure 5b) and 0.7% (marked as (B2) in Figure 5b). The lower loading rate in the $4.4\times 10^{-5}$ $s^{-1}$ experiment reduces matrix hardening, allowing the fibers to carry more load and keep the interfaces intact [72]. This sort of damage evolution and load distribution contribute toward the generation of intralaminar failure mode within the $0^{\circ }$ plies, labeled in (B3) of Figure 5. The increased quasi-static strain rate ($3.6\times 10^{-3}$ $s^{-1}$) qualitatively shows more interfacial damage (mainly interlaminar), and early formation of fiber kinking on the outer edge of the plies. However, the increased loading rate introduces matrix toughening which helps the sample to sustain a relatively higher strain magnitude. The loading applied on the sample is stopped at the machine head displacement of 0.6 mm allowing a complete deformation of the sample. (A4) and (B4) from Figure 5 show the fully deformed sample, convinced by the propagation of the inter-fiber failure within the centrally placed $90^{\circ }$ plies.

The inter-fiber failure (IFF) fracture angle in the transverse compression is reported in the literature through extensive off-axis experimental testing of unidirectional composites [45,73,74]. In this current study, the observed fracture angle in quasi-static and high strain rate experiments are indicated by the blue arrows with magnitude inserted in each of the images containing fully deformed frames of the samples. The inter-fiber failure-driven fracture angles across the strain rates are also summarized in Table 2 along with the data reported in the literature [36,45,74,75]. The fracture angle was observed to be an independent property of the increasing strain rate, as previously reported in [75,76]. The interplay between interlaminar, intralaminar, and fiber kinking mechanisms is crucial in understanding damage propagation in the quasi-isotropic composite. At lower quasi-static strain rates, both interlaminar and intralaminar cracks were observed simultaneously, with intralaminar failure occurring within the $0^{\circ }$ plies (Figure 5). This co-occurrence highlights the distribution of load and the activation of multiple failure modes even at lower strain rates. As the strain rate increases, interlaminar damage becomes more pronounced initially, driven by the weaker ply interfaces. Fiber kinking and intralaminar cracks emerge later in the loading process, reflecting the progressive nature of damage evolution under higher dynamic loads. These observations underline the complex failure behavior of the composite and its sensitivity to strain rate.

**Figure 6:**
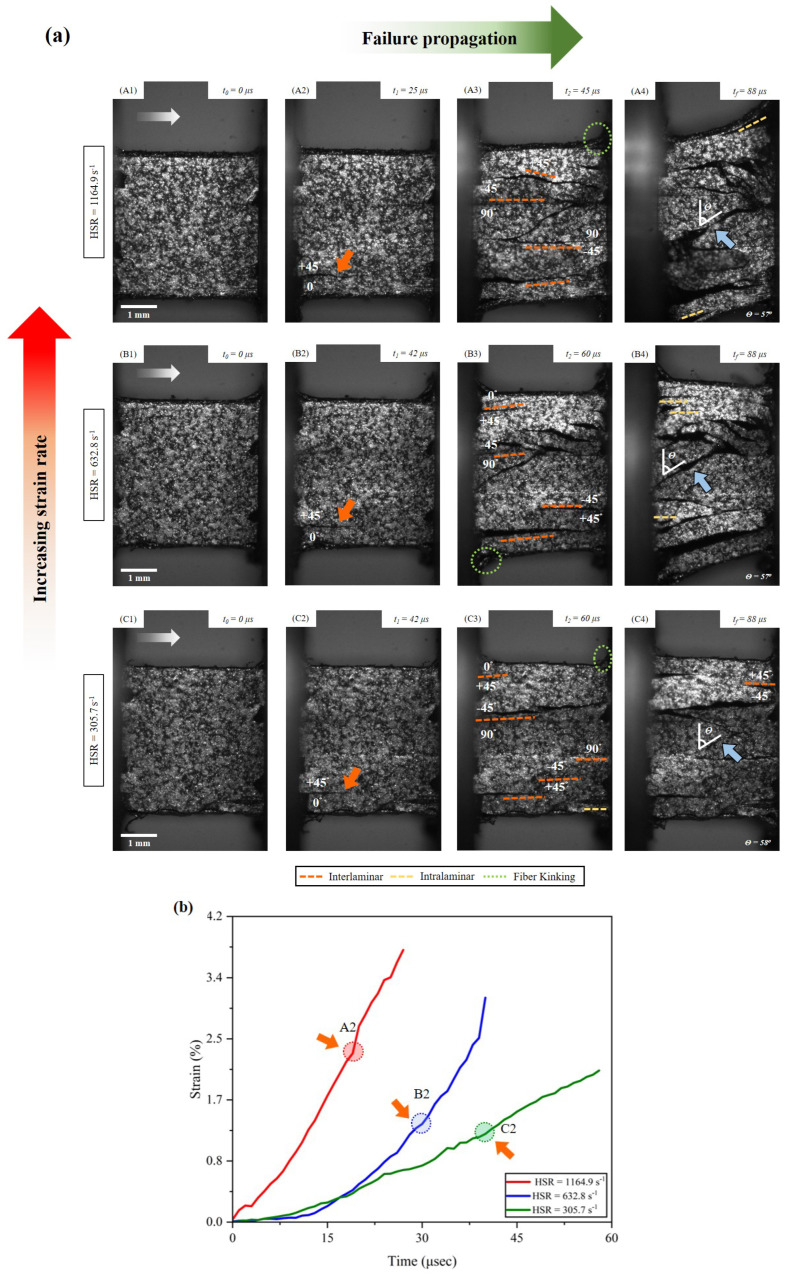
(**a**) Failure propagation in quasi-isotropic carbon-fiber-reinforced polymer composite under high strain rate (HSR) loading (rates denoted in the figure) using a split-Hopkinson Pressure Bar. (**b**) Depicts when crack initiates at different loading rates on the strain-time plot. The white arrow in (**A1**), (**B1**), and (**C1**) indicates the loading direction for compression. Consistent with the QSR results, the primary crack initiates at the interface of $0^{\circ }/45^{\circ }$, as shown in (**A2**), (**B2**), and (**C2**). The fracture plane angle (marked with blue arrows in (**a**)) developed due to inter-fiber failure resulting from the secondary cracks triggered at the $90^{\circ }/-45^{\circ }$ and $+45^{\circ }/-45^{\circ }$ interfaces. The damage in the sample increases with increasing strain rate. The outer $0^{\circ }$ plies experienced fiber kinking, as labeled in (**A3**), (**B3**), and (**C3**). (**A4**), (**B4**), and (**C4**) shows the final recorded deformation of the sample with an indication of the formation of fracture planes.

Figure 6 shows the failure propagation under three ranges of high strain rate experiments. The strain-time plot (Figure 6b) depicts the increasing strain experienced by the sample with the increased strain rates. Also, the images from the ultra-high-speed camera correlated with the strain-time plot to indicate the failure initiation. The mapping of failure initiation on the strain-time plot for different strain rate cases indicates the transition in the matrix behavior as the strain rate increases from 305.7 $s^{-1}$ to 1164.9 $s^{-1}$[77,78]. Similar to the quasi-static results, the failure initiates at the $0^{\circ }$/$+45^{\circ }$ interface for all the high strain rate results. As the sample experiences the propagation of the first crack through $0^{\circ }$/$+45^{\circ }$ interface, secondary cracking occurs at the $90^{\circ }$/$-45^{\circ }$ interface, or at the $+45^{\circ }$/$-45^{\circ }$ interface. The splitting of the sample through $90^{\circ }$/$-45^{\circ }$ triggers the formation of inter-fiber failure in the $90^{\circ }$ plies through the microcracks in the sample. The fracture planes resulting from the loading are indicated by blue arrows and are quantified in Table 2. The main interfacial failure mode is observed to be interlaminar, followed by fiber kinking in the higher strain rates (632.8 and 1164.9 $s^{-1})$ and intralaminar at 305.7 $s^{-1}$. Qualitatively, it can be observed that the sample experiences more damage with the increased strain rate but the damage is more concentrated over the different interfaces. As shown in Figure 6a (A4) and (B4), more damage within the plies can be observed from frame (B4) than (A4). This indicates intralaminar failure dominating once the sample loses its structural integrity.

**Figure 7:**
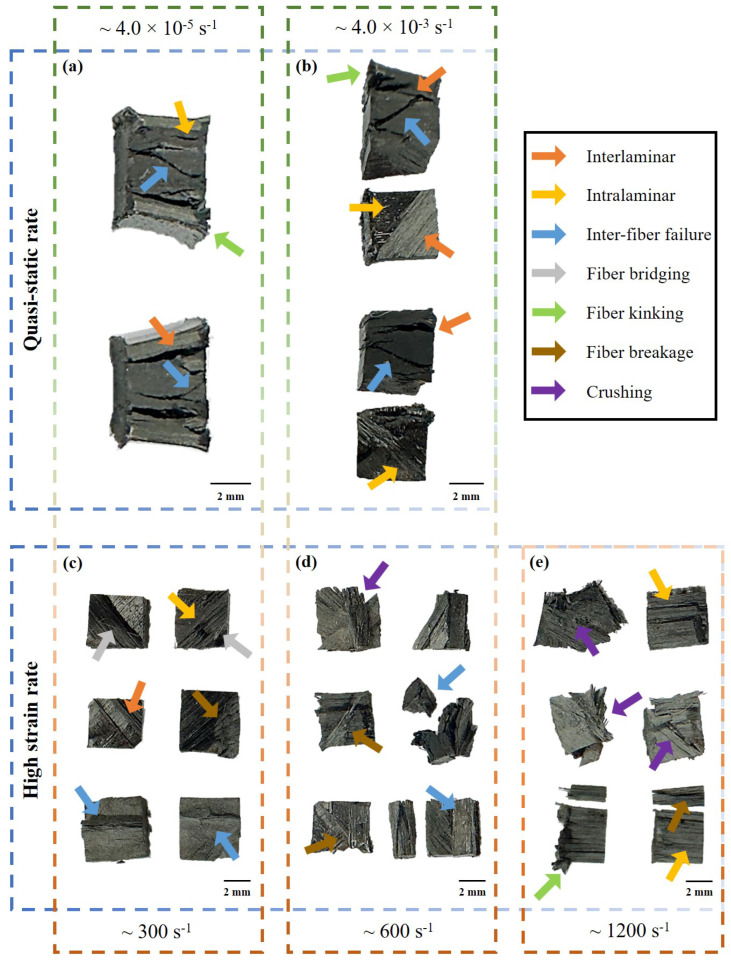
Damage morphology of the quasi-isotropic composite under different quasi-static (**a**,**b**) and high strain rate (**c**–**e**) ranges. The damage increases with strain rates and depicts the typical failure modes and features of fiber-reinforced composites. (**a**,**b**) demonstrate the propagation of cracks majorly from the interlaminar interface between $0^{\circ }$/$45^{\circ }$. (**c**–**e**) shows fragments from the high strain rate testing under in-plane compression loading. The inter-fiber failure fracture plane can be observed in the quasi-static and high strain rate experiments.

The failure modes and features, deformed samples, and fragments from the experiments are presented in Figure 7. As concluded from the images, the sample demonstrates the generation of fiber kinking at the $0^{\circ }$ plies under both quasi-static and high strain rate loading. The samples remain intact and only split at the interfaces (due to the shearing effect between the plies) until ∼300 $s^{-1}$. The formation of the fracture plane in the samples can be seen in Figure 7a,b,d. Over the higher strain rates (∼600 –∼1200 $s^{-1})$, the sample is believed to experience crushing causing fibers of different orientations to infuse within each other. Overall, the sample has interfacial failure propagation as shown by the split adjacent layers. Finally, Figure 7e shows the fragmented plies and evidence of crushing and intralaminar failure at 1164.9 $s^{-1}$.

**Figure 8:**
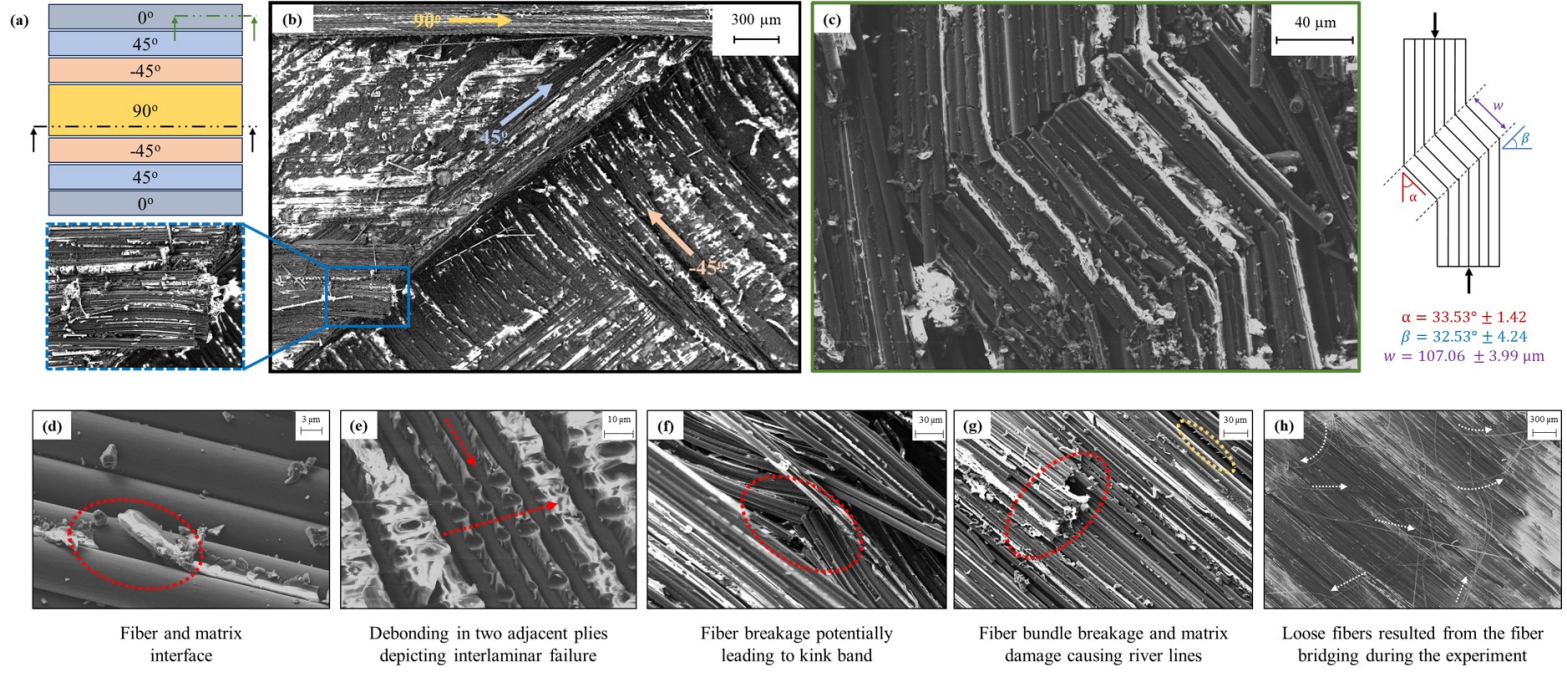
Fracture surface mapping on failure samples and fragments from a quasi-isotropic carbon-fiber-reinforced polymer composite. The ply sequence schematic is shown in (**a**) and a realistic global map of the different fiber orientations involved in the sample is shown in (**b**). Fiber micro-buckling, also known as fiber kinking, is shown in (**c**), demonstrating the bending of fibers, loaded by the matrix in shear, along with the quantification of the orientation angle (β), inclination angle (α), and kink band with (*w*). (**d**,**e**) indicates the localized debonding between the fiber and matrix interfaces, and between two adjacent plies dominated by shear loading. Typical fiber failure modes are observed in (**f**–**h**). Interestingly, (**h**) showcases loose fibers potentially resulting from the fiber bridging that occurred during the longitudinal compressive loading.

The fracture surface mapping was conducted using SEM on the fragments of the sample from high strain rate experiments, as shown in Figure 8. The figure shows the different failure mechanisms observed at different locations, as marked in Figure 8a and the global map in Figure 8b to indicate the complex failure modes with different fiber orientations. Figure 8c depicts the formation of the kink band repercussion of the fiber kinking. The formation of the fiber kinking is a result of the micro-buckling of the fibers when the load is applied with the alignment of their axis [79]. The kink band can be quantified based on the orientation angle (β), inclination angle (α), and kink band width (w)[80]. In their study, Wadee et al. [81] provide insights into the correlation and influence of defining parameters of kink band formed in unidirectional composite laminate with sequential propagation and driving mechanisms for (α) and (β) at; (α=0), (α=β), (α=2β), and (α>β). In the present work, the reported kink band orientation angle $\left(\beta =32.53^{\circ }\pm 4.24^{\circ }\right)$, inclination angle $\left(\alpha =33.53^{\circ }\pm 1.42^{\circ }\right)$, and kink band width (w=107.06±3.99μm) are consistent with the magnitude reported by [82,83,84,85]. This shows that the mechanisms are independent of the stacking sequence of the composite and the magnitude of the angles associated with kink does not change. Figure 8d–e shows the matrix-driven failure features due to shear dominance, debonding between the fiber and matrix interface, and two adjacent plies. Fiber buckling, fiber bundle breakage, fiber pull-out, fiber debonding, and fiber bridging are shown in Figure 8f–h. The compressive loading with the alignment of the fiber orientation causes the bulging formation which either leads to kink band or fiber bundle breakage [86,87]. Fiber bridging induces additional toughness in layered unidirectional composites by delaying the delamination growth [88,89]. The loose fibers reported in Figure 8h are evident for the fiber bridging phenomenon happening between the $0^{\circ }$ and $45^{\circ }$ plies during dynamic compression loading.

### 3.3. Crack Speed Measurements

**Figure 9:**
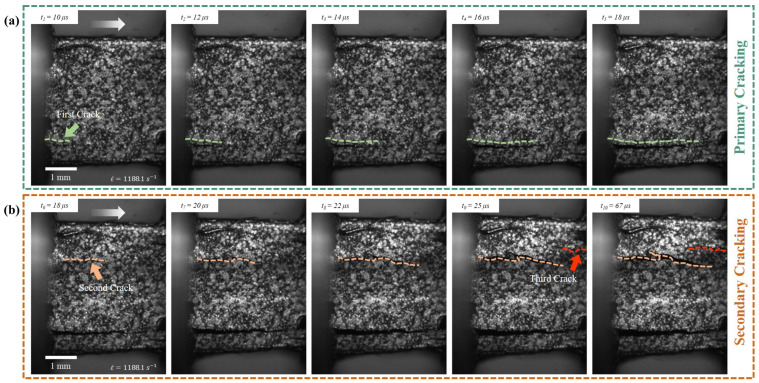
Crack length tracing methodology of the first, second, and third cracks developed at 1188.1 $s^{-1}$. The cracks are grouped as (**a**) primary cracking (first crack), and (**b**) secondary cracking, which includes both the second and third cracks developed in the sample. The white arrow indicates the direction of compression loading.

**Figure 10:**
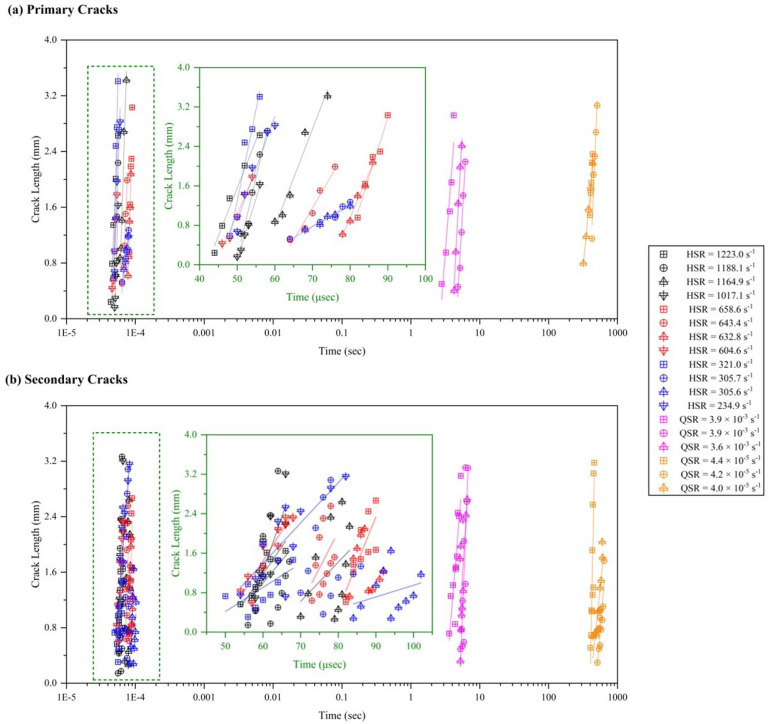
Plot shows the length of the crack measured for each case. For reliable quantification of the (**a**) primary cracks and (**b**) secondary cracks, at least five sets of frames are considered for calculating the crack speed for each case. The slope generated for quasi-static and high strain rates determines the average crack speed.

In this section, the crack speed measurements at different strain rates are presented. The cracking developed during the experiments can be categorized as primary or secondary cracking, and based on the interfaces involved (inter- and intralaminar cracks). Figure 9 shows a failure propagation at 1188.1 $s^{-1}$ with marking to illustrate the full development of the first crack (termed primary cracking), and the second and third cracks (termed secondary cracking). The length of each of these cracks is measured using ImageJ 1.54i, 3 March 2024 [90] software. The crack growth event for each case was quantified manually by selecting at least five sets of frames at 1 million frames-per-second, as shown in Figure 10. The average crack speed for primary cracks increases from 0.018 mm/s (∼ $4.0\times 10^{-5}$ $s^{-1}$) to 1.574 mm/s (∼ $4.0\times 10^{-3}$ $s^{-1}$) within quasi-static strain rates. The trend of increasing crack speed with increased strain rates continues in the high strain rate experiments as well.

**Figure 11:**
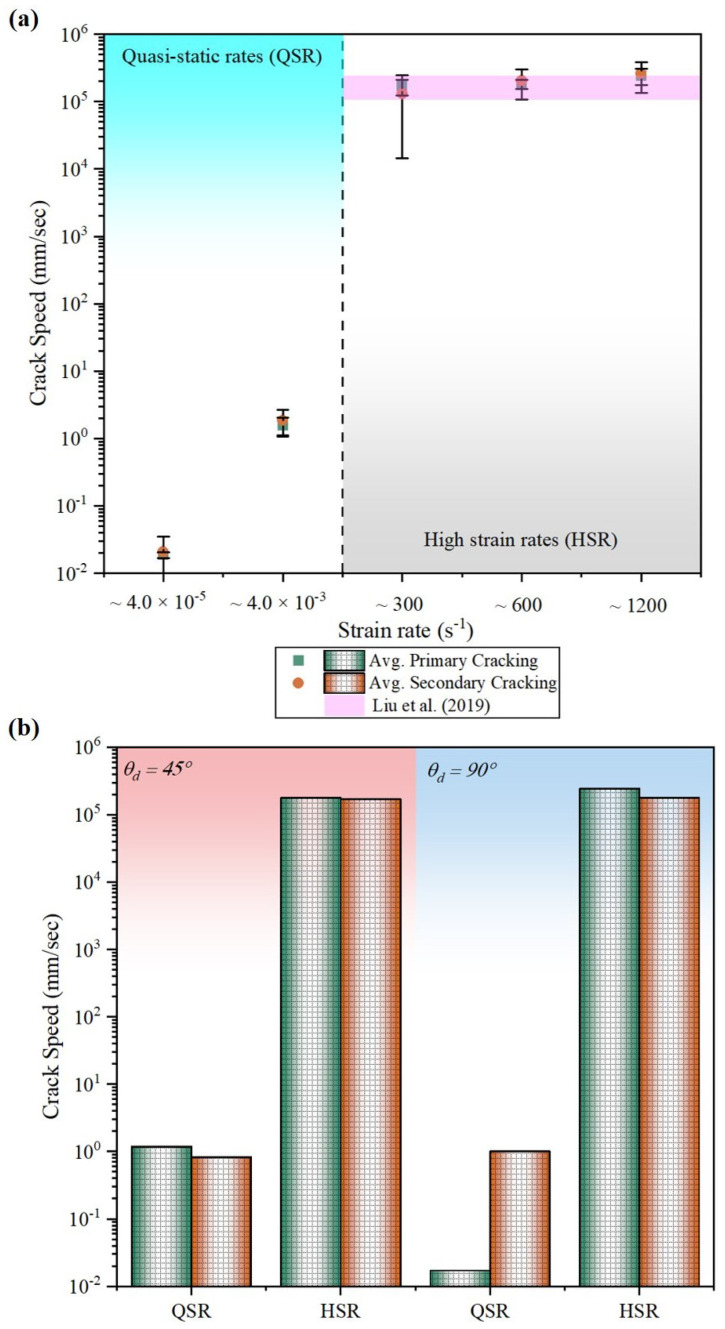
Plot of (**a**) average primary and secondary crack speed at the different ranges of strain rates considered in this study. (**b**) the variation in average crack speed at different interfaces defined by the relative angle $\left(\theta_{d}\right)$ between the participating adjacent plies during interlaminar crack propagation. The presented data are compared with the high strain rate experimental data from Liu et al. [91].

The average crack speed for primary cracks in the high strain rate experiments increases from $1.67\times 10^{5}$ (measured for ∼300 $s^{-1}$ mm/s) to $2.42\times 10^{5}$ mm/s (measured for ∼1200 $s^{-1}$). The average crack speed at ∼600 $s^{-1}$, calculated as $1.82\times 10^{5}$ mm/s, is consistent with the double-cantilever beam (DCB) experimental data on carbon-epoxy composite reported by Liu et al. [91]. The secondary crack speed is found to be more than the primary crack as the sample has already lost some structural integrity. Figure 11a summarizes the crack speed for primary and secondary across the strain rate. The crack speed depends on the fiber orientation of the composite material, as the shearing effect will be influenced by the relative angle $\left(\theta_{d}\right)$ between the adjacent plies participating in the crack propagation [91]. Figure 11b shows the crack speed based on the fiber orientation involved in the interlaminar crack propagation. The average crack speed reduces with the increase in the relative angle $\left(\theta_{d}\right)$ of the interfaces at the quasi-static strain rate. However, there is no significant difference in the high strain rate tests which can account for the variability from the manual procedure employed in this study. The crack speed for the primary cracking at the $0^{\circ }$/$+45^{\circ }$ and $90^{\circ }$/$-45^{\circ }$ turns out to be 1.2 mm/s for quasi-static strain rate and $1.78\times 10^{5}$ mm/s at high strain rates. The secondary crack speed for the same experiment is profoundly reduced by 33% at quasi-static strain rates but minimal reduction (4%) in high strain rate experiments is observed. The strongest interlaminar bonding in the quasi-isotropic composite is found to be at the $+45^{\circ }$/$-45^{\circ }$ interface $\left(\theta_{d}=90^{\circ }\right)$ with a minimum magnitude of 0.017 mm/s through all the cases presented in this paper.

### 3.4. Dynamic Constitutive Model: ZWT

The stress-strain plots from the experiments establish the strain-rate-dependent behavior of the material and non-linear damage evolution under high strain rate loading. A modified version of the viscoelastic model proposed [53,92,93] is utilized to better describe the dynamic stress-strain and compressive behavior of the quasi-isotropic carbon/epoxy composite considered in this study. The model contains a high-frequency Maxwell’s element and a nonlinear elastic spring in parallel to characterize the high strain rate response of the material [56,94]. The model can be expressed as [95,96]

(4) $$\sigma =f\left(\varepsilon \right)+E_{2}\int_{0}^{t}\dot{\varepsilon }\left(\tau \right)exp\left(-\frac{t-\tau }{\theta }\right)d\tau$$

(5) $$f\left(\varepsilon \right)=E_{1}\varepsilon +\alpha \varepsilon^{2}+\beta \varepsilon^{3}$$

(6) $$\sigma =E_{1}\varepsilon +\alpha {\left(\dot{\varepsilon }\right)}^{2}+E_{2}\theta \left[1-exp\left(-\frac{\varepsilon }{\theta \dot{\varepsilon }}\right)\right]$$

where f(ε) defines the nonlinear elastic equilibrium and parameters $E_{1},$ α, and β account for the elastic constants. $\dot{\varepsilon }$ is the measured constant strain rate experienced by the sample in high strain rate experiments. The integral component of Equation (4) corresponds to the high-frequency Maxwell’s element, and ε and σ are the Cauchy stress and strain parameters, respectively. θ accounts for the relaxation time and $E_{2}$ is the elastic constant included in the model to represent the relaxation time for Maxwell’s element. Based on the assumption of constant strain rate attained in the sample and the brittle nature of the material (very small failure strain), the cubic term $\left(\beta \varepsilon^{3}\right)$ can be neglected without affecting the prediction accuracy of the model [63]. Equations (4) and (5) can be simplified into.

**Figure 12:**
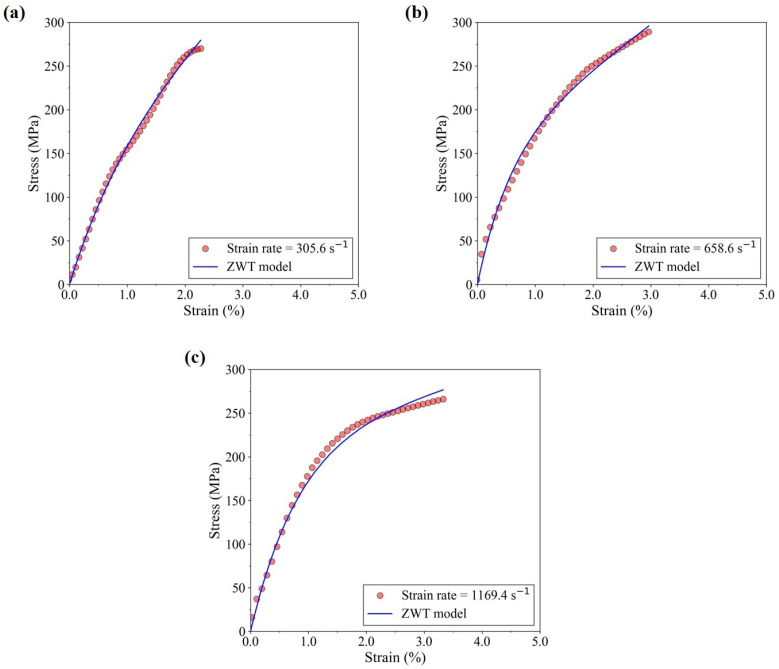
Experimental and ZWT-fitted stress-strain curves for the quasi-isotropic composite under high strain rates (**a**) 305.6 $s^{-1}$, (**b**) 658.6 $s^{-1}$, and (**c**) 1169.4 $s^{-1}$.

Figure 12 shows the comparison of the ZWT-fitted stress-strain plots at three high strain rates with the experimentally obtained plots. The optimal parameters were obtained through curve fitting using Python scripting, and are summarized in Table 3. The good fit of the predicted plots to the experimental data obtained through constitutive modeling establishes the viability of implementing a viscoelastic model to characterize the high strain rate response of the studied composite.

## 4. Conclusions

This paper studied the strain-rate-dependent behavior of a quasi-isotropic $[0^{\circ }/\pm$ $45^{\circ }$/$90^{\circ }{]}_{s}$ carbon-fiber-reinforced polymer composite laminate under in-plane compressive loading. Utilizing T700SC carbon fibers and toughened thermoset epoxy resin MTC510, the composite samples were prepared through an autoclave curing process, ensuring a fiber volume fraction of 60% and a cured ply thickness of 0.28 mm. Experiments conducted across a spectrum of strain rates, from quasi-static to high strain rates, were supported by the use of advanced imaging techniques, including digital image correlation and scanning electron microscopy. This allowed for the detailed observation of the deformation mechanisms and failure modes to reveal the interplay of different interlaminar and intralaminar failure modes (such as fiber kinking, inter- and intralaminar cracking, and matrix-driven failures).

The experiments demonstrated a clear strain-rate dependency in the compressive strength and failure strain of the composite, with both metrics increasing as the strain rate rose. Digital image correlation facilitated the measurement of strain fields, enabling detailed observation of complex deformation and failure patterns. Post-mortem analyses using scanning electron microscopy revealed prominent failure mechanisms, including fiber kinking, matrix cracking, and both interlaminar and intralaminar fractures. These analyses highlighted that intralaminar fracture planes, forming at angles between $52^{\circ }$ and $58^{\circ }$, remained consistent across varying strain rates, signifying their independence from the loading rate. Crack propagation speeds were observed to escalate with increasing strain rates, with primary cracks initiating along fiber interfaces and secondary cracks emerging as structural integrity degraded. Crack speeds also exhibited strong dependency on fiber orientation, further emphasizing the interplay between material architecture and loading conditions. Finally, the Zhu–Wang–Tang model reliably characterized the composite’s non-linear viscoelastic behavior under dynamic loading, providing a robust framework for predicting performance in extreme conditions. These findings collectively deepen the understanding of the failure behavior and dynamic response of carbon-fiber-reinforced polymer composites, crucial for their application in high-performance environments.

The observed strain-rate-dependent behavior of the quasi-isotropic CFRP composite has significant implications for applications in aerospace, automotive, and defense industries, where materials are subjected to dynamic loading conditions. These findings can guide material selection and design strategies for impact-resistant and lightweight structures. Future studies could extend this work by investigating alternative layup configurations, hybrid composites, or the influence of environmental factors, such as temperature and humidity. These efforts would provide a more comprehensive understanding of the material’s performance under diverse real-world conditions, further advancing the field of composite materials engineering.

## Figures and Tables

**Table 1:** Summary of the experiments performed for in-plane compression under quasi-static and dynamic loading conditions and obtained mechanical response for the quasi-isotropic composite material.

Experiment Type	Conditions	Strain Rate ($s^{-1}$)	In-Plane Compression Strength (MPa)	Stiffness (MPa)	Failure Strain
Quasi-Static	Loading rate $4\times 10^{-4}$ mm/s	$4.4\times 10^{-5}$	131.6	32,317	0.008
		$4.3\times 10^{-5}$	126.0	31,652	0.007
		$4.2\times 10^{-5}$	132.6	22,990	0.008
	Loading rate $4\times 10^{-2}$ mm/s	$3.6\times 10^{-3}$	166.3	38,187	0.010
		$3.9\times 10^{-3}$	191.5	38,513	0.008
		$3.9\times 10^{-3}$	190.4	38,041	0.008
Dynamic	Pulse shaper = HDPE Pressure = 25 psi	305.6	270.0	16,972	0.022
		305.7	258.3	16,522	0.021
		321.0	224.5	16,621	0.019
	Pulse shaper = HDPE Pressure = 40 psi	632.8	275.2	19,795	0.025
		643.4	227.8	19,038	0.013
		658.6	289.2	19,607	0.029
	Pulse shaper = Paper Pressure = 40 psi	1169.4	259.6	19,270	0.028
		1188.1	259.2	19,861	0.032
		1198.0	247.9	19,497	0.290

**Table 2:** Fracture angle obtained at different strain rates.

	Strain Rate	Fracture Angle (θ)
	QSR = $4.4\times 10^{-5}$ $s^{-1}$	$55^{\circ }$
	QSR = $3.6\times 10^{-3}$ $s^{-1}$	$52^{\circ }$
Present study	HSR = 305.7 $s^{-1}$	$58^{\circ }$
	HSR = 632.8 $s^{-1}$	$57^{\circ }$
	HSR = 1164.9 $s^{-1}$	$57^{\circ }$
Puck et al. [36]	QSR	$53^{\circ }$
Vural et al. [75]	QSR	$53^{\circ }$
Koeber et. al [45]	HSR	$56^{\circ }$
Thomson et al. [74]	HSR	$58^{\circ }$

**Table 3:** Parameters from ZWT constitutive model for quasi-isotropic carbon-fiber-reinforced composites.

Strain Rate $\left(s^{-1}\right)$	$E_{1}$ (GPa)	$E_{2}$ (GPa)	θ (μs)	α (GPa)
305.6	5.7	15.5	0.34	10.0
658.6	4.4	27.2	8.8	10.0
1169.4	1.0	27.2	7.4	11.5

## Data Availability

The data presented in this study are available on request from the corresponding author. Due to privacy and the nature of the project, the data is not available online.
